# A Study on the Correlation Between Calcific Aortic Valve Disease and Carotid Artery Elasticity

**DOI:** 10.31083/RCM26821

**Published:** 2025-05-21

**Authors:** Yan Zhang, Hui Wang, Yu Zhong, Wei Wang, Zhijun Zhang, Quan He

**Affiliations:** ^1^Department of Ultrasound, University-Town Hospital of Chongqing Medical University, 401331 Chongqing, China; ^2^Department of Cardiology, The First Affiliated Hospital of Chongqing Medical University, 400016 Chongqing, China

**Keywords:** ultra-fast pulse wave technology, calcified aortic valve disease, carotid artery elasticity

## Abstract

**Background:**

This study aimed to investigate the correlation between calcific aortic valve disease (CAVD) and carotid artery elasticity using ultra-fast pulse wave velocity (UFPWV) technology. Early detection of alterations in carotid artery elasticity, coupled with the prompt implementation of intervention strategies, can effectively decrease the incidence of cardiovascular diseases.

**Methods:**

Patients with CAVD were recruited from the University-Town Hospital of Chongqing Medical University and placed in the observation group. Meanwhile, an equivalent number of patients with non-calcified aortic valve disease were recruited as controls. All participants underwent comprehensive health assessments, including measurements of blood lipids, fasting blood sugar, and other biochemical indicators. Additionally, bilateral carotid intima-media thickness (CIMT) was measured, as well as pulse wave velocity (PWV) at the beginning of systole (PWV-BS) and the end of systole (PWV-ES). Differences in various indicators between the two groups were analyzed, and the factors associated with CAVD and carotid artery elasticity were investigated. The correlation between CAVD and carotid artery elasticity was also evaluated.

**Results:**

Patients with CAVD exhibited significantly higher CIMT, PWV-BS, and PWV-ES levels than those with non-calcified aortic valve disease (*p* < 0.01). PWV-BS and PWV-ES showed progressive increases according to the severity of calcification. Coronary atherosclerotic heart disease and PWV-BS were all identified as independent risk factors for CAVD. The risk factors associated with PWV-BS include hypertension, coronary atherosclerotic heart disease, total cholesterol, and homocysteine (*p* < 0.05 for all). The risk factors related to PWV-ES include hypertension, coronary atherosclerotic heart disease, total cholesterol, and glycated hemoglobin (*p* < 0.05 for all).

**Conclusions:**

UFPWV technology is a novel method for the early diagnosis of carotid elasticity. Evaluating carotid artery atherosclerosis in patients with CAVD may lead to earlier detection and intervention and reduce the incidence of cardiovascular events.

## 1. Introduction

The most common etiology of aortic stenosis (AS) is calcified aortic valve disease (CAVD) [1]. The calcification of cardiac valves and atherosclerosis show similar alterations in tissue pathology [2]. The number of CAVD cases reported among younger patients has recently increased [3]. Carotid atherosclerosis indicates the occurrence and progression of arterial atherosclerotic lesions throughout the body [4]. The 2018 guidelines for hypertension management by the European Society of Hypertension and the European Society of Cardiology highlight pulse wave velocity (PWV) as the gold standard for assessing arterial stiffness due to its direct correlation with Young’s modulus [5]. The significance of using ultra-fast pulse wave velocity (UFPWV) technology in assessing carotid artery elasticity has previously been well-established [6]. However, the application of changes in carotid artery elasticity in patients with CAVD is currently limited, and there is a paucity of research on this topic. Therefore, the present study utilized UFPWV technology to assess the elasticity of the carotid artery. We then investigated the association between CAVD and alterations in carotid artery elasticity. Identifying the risk factors for CAVD and carotid artery elasticity has significant clinical importance for risk stratification and managing cardiovascular diseases.

## 2. Materials and Methods

### 2.1 Study Design

This single-center, retrospective study evaluated 105 patients diagnosed with CAVD who were admitted to the University-Town Hospital of Chongqing Medical University between September 2022 and February 2024. In addition, 105 healthy subjects were recruited from our hospital during the same period as controls.

### 2.2 Inclusion and Exclusion Criteria

Patients diagnosed with CAVD by echocardiography were informed about the study, and those who agreed to participate signed an informed consent. Patients with a diagnosis of carotid artery atherosclerotic plaques, as characterized by an intima-media thickness (IMT) of ≥1.5 mm at any location along the carotid artery, a protrusion into the vascular lumen, or localized thickening exceeding 50% of the surrounding IMT, were excluded [7]. Patients were also excluded if they possessed congenital heart diseases, had undergone heart valve replacement surgery, had pulmonary disease, rheumatic heart disease, infective endocarditis, syphilis, or other related conditions. Since this type of disease can directly damage the heart valves, the extent of valve calcification can be difficult to monitor. Thus, patients who did not cooperate with the examination were also excluded.

### 2.3 CAVD Assessment

The ACUSON Redwood (Serial No. 561045. Healthineers SIEMENS, Seongnam-si, Gyeonggi-do, Korea) with a cardiac probe (2–4 MHz) was used in this study as the cardiovascular imaging ultrasonic diagnostic instrument.

Two experienced ultrasound doctors performed standardized echocardiograms in accordance with the 2022 British Society of Echocardiography (BSE) Practice Guidelines [8]. Enhanced echo and leaflet stiffness of the aortic valve and a valve thickness exceeding 3 mm were applied to indicate a diagnosis of CAVD. The extent of aortic valve calcification was assessed. Subsequently, the subjects were categorized into three groups based on the severity of calcification: Group One, mild calcification of the aortic valve, characterized by the presence of calcification but occupying no more than one-third of the leaflet area; Group Two, moderate calcification of the aortic valve, where the extent of calcification covered less than two-thirds of the valve surface area; Group Three, severe calcification involving more than two-thirds of the leaflet area.

### 2.4 Clinical Data

The medical staff collected all clinical data pertaining to the study population. This study encompassed several fundamental health metrics, including body mass index (BMI) and biochemical parameters such as blood lipids, and also documented the comorbidity profiles of participants. The diagnostic criteria for hypertension in patients were as follows: In the absence of antihypertensive medication, a clinical blood pressure exceeding 140/90 mmHg; home blood pressure exceeding 135/85 mmHg; or 24 h ambulatory blood pressure readings exceeding 130/80 mmHg, with daytime readings exceeding 135/85 mmHg and night-time readings exceeding 120/70 mmHg. The diagnostic criteria for hypertension were derived from the 2024 “Guidelines for the Management of Hypertension and Elevated Blood Pressure” issued by the European Society of Cardiology (ESC) [9]. The diagnostic criteria for diabetes were fasting blood glucose ≥7.0 mmol/L, random blood glucose ≥11.1 mmol/L, or 2 h blood glucose in the Oral Glucose Tolerance Test (OGTT) ≥11.1 mmol/L. The diagnostic criteria for diabetes were established based on the 2024 “Guidelines for the Diagnosis and Classification of Diabetes” [10]. Coronary atherosclerotic heart disease (CHD) is defined as a condition identified in patients through coronary angiography or cardiac computed tomography angiography (CTA). The diagnostic criteria for CHD were based on the 2024 ESC “Guidelines for Managing Chronic Coronary Syndromes” [11].

### 2.5 Carotid Intima-media Thickness (CIMT) and PWV Measurements

Two physicians with at least 10 years of experience conducted the carotid artery ultrasound examinations per the 2017 ESC “Guidelines for Diagnosing and Treating Peripheral Arterial Diseases” [12] and standardized carotid PWV measurements using ultrafast ultrasound imaging. After completing the pertinent training and subsequent evaluations, two physicians assessed the carotid arteries of patients without knowledge of their overall clinical indicators.

The participants underwent carotid ultrasound imaging in the supine position using the Aixplorer ultrasound system (Serial No. IQ78PR61. SuperSonic lmagine, Les Jardins de la Duranne, BÃt E et F, 510, rue René Descartes, Aix-en-Provence, France), equipped with a linear array probe (4–15 MHz and utilizing the vascular PWV mode). The measurement technique for CIMT and carotid artery PWV was described in an earlier multicenter study conducted by Lixue Yin *et al*. [13]. The subject reclines and rests for 15 minutes before fully exposing the neck. The probe was positioned along the outer border of the sternocleidomastoid muscle to capture a longitudinal section of the carotid artery spanning from the proximal to the distal end and ensuring continuous display of the IMT. The IMT of the posterior wall was measured 1 cm below the bifurcation point of the common carotid artery during diastole. The patient was instructed to hold their breath and initiate PWV once the image had stabilized. The Aixplorer ultrasound system automatically measured the pulse wave velocity at the beginning of systole (PWV-BS) and pulse wave velocity at the end of systole (PWV-ES). The system automatically computed the variance in the PWV measurements during the beginning of systole (BS) and end of systole (ES), reported as Δ±. In this study, the Δ± values were regulated to be <20% of the PWV values during BS and ES. If the value was ≥20%, the PWV was remeasured. All measurements were performed three times from each side of the common carotid artery, and the mean values were calculated. The measurements for both PWV-BS and PWV-ES were the average values of both sides.

### 2.6 Statistical Analysis

Measurement data were analyzed using the SPSS 27.0 statistical software package (SPSS Software version 27, IBM Corp., Chicago, IL, USA). Quantitative data were subjected to the PWV normal distribution test. Qualitative data adopts the chi-square test. Data are expressed as the mean ± standard deviation, with the *t*-test performed in accordance with the normal distribution. For non-normally distributed quantitative data, the Mann-Whitney U test is employed. When data adhered to the normal distribution, analysis of variance (ANOVA) was utilized to conduct multiple group comparisons. Multiple linear regression analysis was used to analyze the risk factors for carotid artery PWV. Stepwise linear regression analysis was performed to identify statistically significant variables. These were subsequently integrated into the multiple linear regression model for a more comprehensive analysis. Using the degree of CAVD as the dependent variable, a value of 0 was designated for patients exhibiting no or mild aortic valve calcification; conversely, a value of 1 was assigned for those with moderate to severe aortic valve calcification. This analysis employed stepwise multivariate binary logistic regression analysis to investigate the factors influencing CAVD. A *p*-value < 0.05 was considered statistically significant.

## 3. Results

### 3.1 Patient Characteristics

No statistically significant differences were observed between the observation group and the Non-CAVD group regarding gender, age, BMI, proportion of smokers, proportion of diabetes patients, and fasting blood glucose levels (all *p* > 0.05). However, the CAVD group exhibited a significantly higher prevalence of hypertension, coronary heart disease, a significantly lower prevalence of Hyperlipidemia compared to the Non-CAVD group (*p* < 0.05). It is significant differences were observed between the observation group and the Non-CAVD group regarding C-peptide, free fatty acids and total cholesterol (*p* < 0.05) (Table 1).

**Table 1.** **Clinical data between Non-CAVD group and CAVD group (n = 210)**.

Variables		Non-CAVD group	CAVD group	*p*-value
		(n = 105)	(n = 105)	
Age (years)		58.0 [54.0; 62.0]	58.0 [56.0; 60.0]	0.622
Gender	Female	48 (45.7%)	45 (42.9%)	0.6771
	Male	57 (54.3%)	60 (57.1%)	
Smoking		25 (23.8%)	38 (36.2%)	0.050
BMI (kg/m^2^)		24.16 ± 3.67	24.77 ± 3.36	0.319
Consolidate disease conditions	Hypertension	41 (39.0%)	65 (61.9%)	<0.001
	Diabetes	40 (38.1%)	44 (41.9%)	0.573
	Coronary atherosclerotic heart disease	8 (7.6%)	24 (22.9%)	0.002
	Hyperlipidemia	21 (20.0%)	10 (9.5%)	0.032
The laboratory examination	HbA1c	5.20 [4.20; 6.70]	5.45 [4.61; 6.87]	0.133
	C-peptide	2.45 [1.83; 3.08]	2.89 [2.36; 3.25]	0.006
	FPG	5.56 [4.79; 7.18]	5.60 [4.75; 7.43]	0.796
	TC (mmol/L)	3.2 [2.49; 4.23]	4.04 [3.19; 4.65]	<0.001
	LDL-C (mmol/L)	2.14 [1.66; 2.68]	2.27 [1.71; 2.93]	0.060
	HDL-C (mmol/L)	1.45 [1.25; 1.65]	1.46 [1.25; 1.62]	0.524
	FFA (mmol/L)	0.46 [0.33; 0.62]	0.58 [0.46; 0.65]	<0.001
	TG (mmol/L)	1.42 [1.25; 1.74]	1.35 [1.02; 1.65]	0.315
	HCY	8.33 [7.60; 9.21]	8.65 [7.29; 10.24]	0.082
	UA	346 [310; 385]	346 [307; 379]	0.915
	TSH	2.15 [1.00; 3.20]	2.05 [1.07; 2.90]	0.816
	CYSC	0.89 [0.75; 1.02]	0.92 [0.81; 1.06]	0.062

Abbreviations: BMI, body mass index; FFA, free fatty acids; TC, total cholesterol; TG, triglycerides; HbA1c, glycated hemoglobin; FPG, fasting blood glucose; HCY, homocysteine; UA, uric acid; CYSC, cystatin C; CAVD, calcific aortic valve disease; LDL-C, low-density lipoprotein cholesterol; HDL-C, high-density lipoproteincholesterol; TSH, thyroid stimulating hormone.

### 3.2 CIMT and PWV Results

**Fig. 1.**
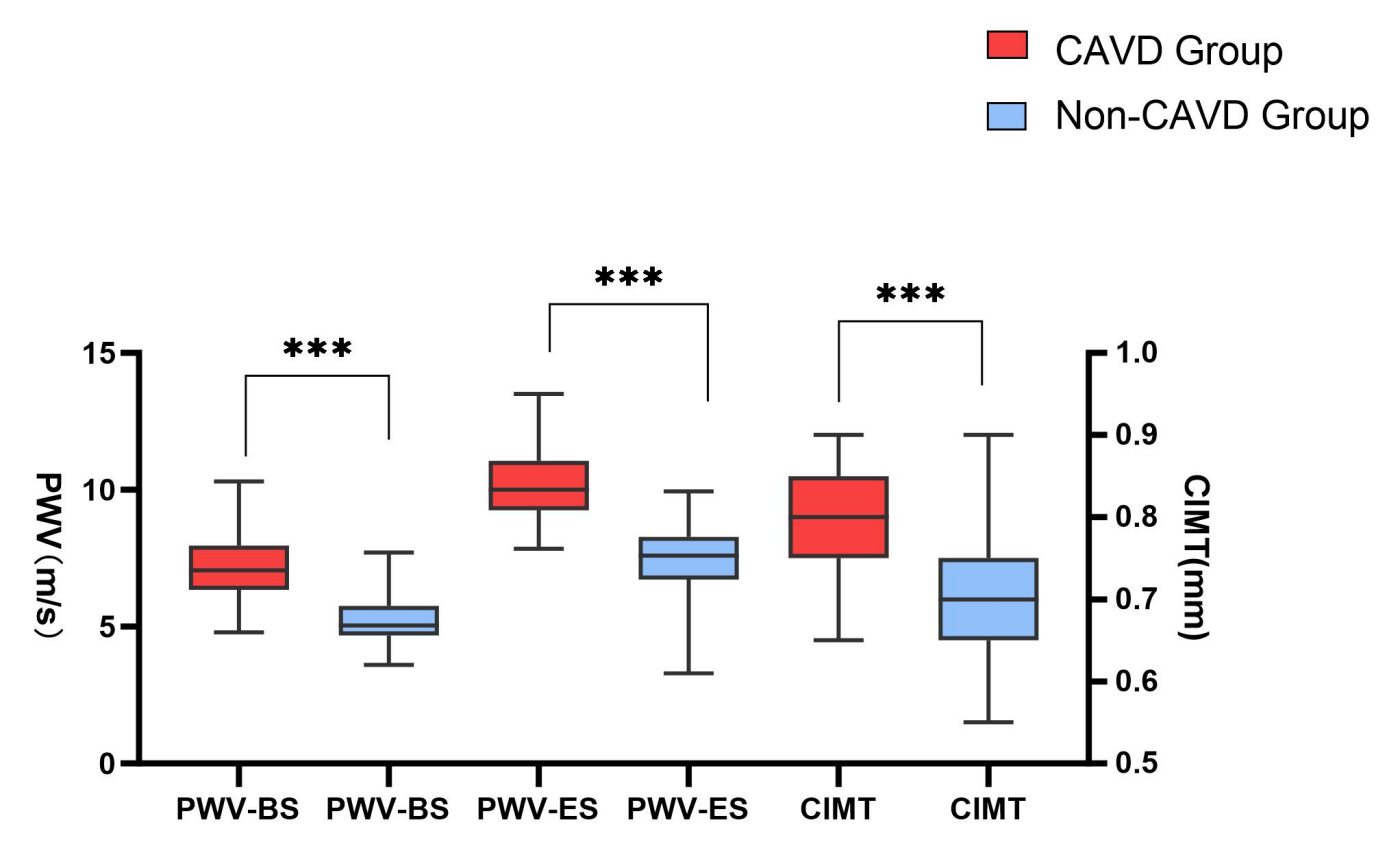
**Boxplot of the carotid intima-media thickness (CIMT) and carotid artery pulse wave velocity (PWV) in the observation and Non-CAVD groups**. The boxplots present interquartile ranges (in mm and m/s) (*** *p* < 0.001). PWV-BS, pulse wave velocity at the beginning of systole; PWV-ES, pulse wave velocity at the end of systole.

**Fig. 2.**
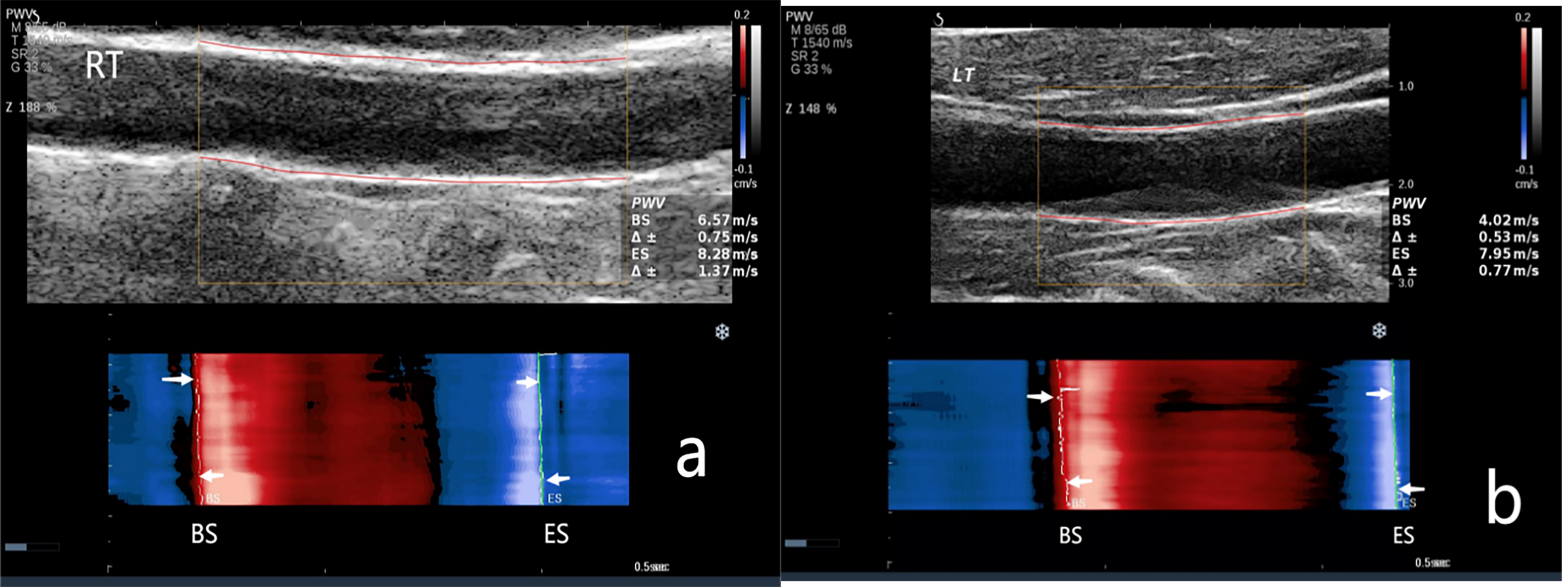
**The carotid artery PWV in the Non-CAVD group**. (a) PWV measurement of the right carotid artery. PWV-BS, 6.57 m/s; PWV-ES, 8.28 m/s. (b) PWV measurement of the left carotid artery. PWV-BS, 4.02 m/s; PWV-ES, 7.95 m/s. NOTE: PWV-BS corresponds to the slope of the most prominent line in the red band, whereas PWV-ES is associated with the slope of the most prominent line in the blue band. These features are highlighted in the figure by the white arrows. M, map; T, tissue tuner; SR, speckle reduction; G, gain; Z, zoom; dB, decibel; BS, beginning of systole; ES, end of systole.

**Fig. 3.**
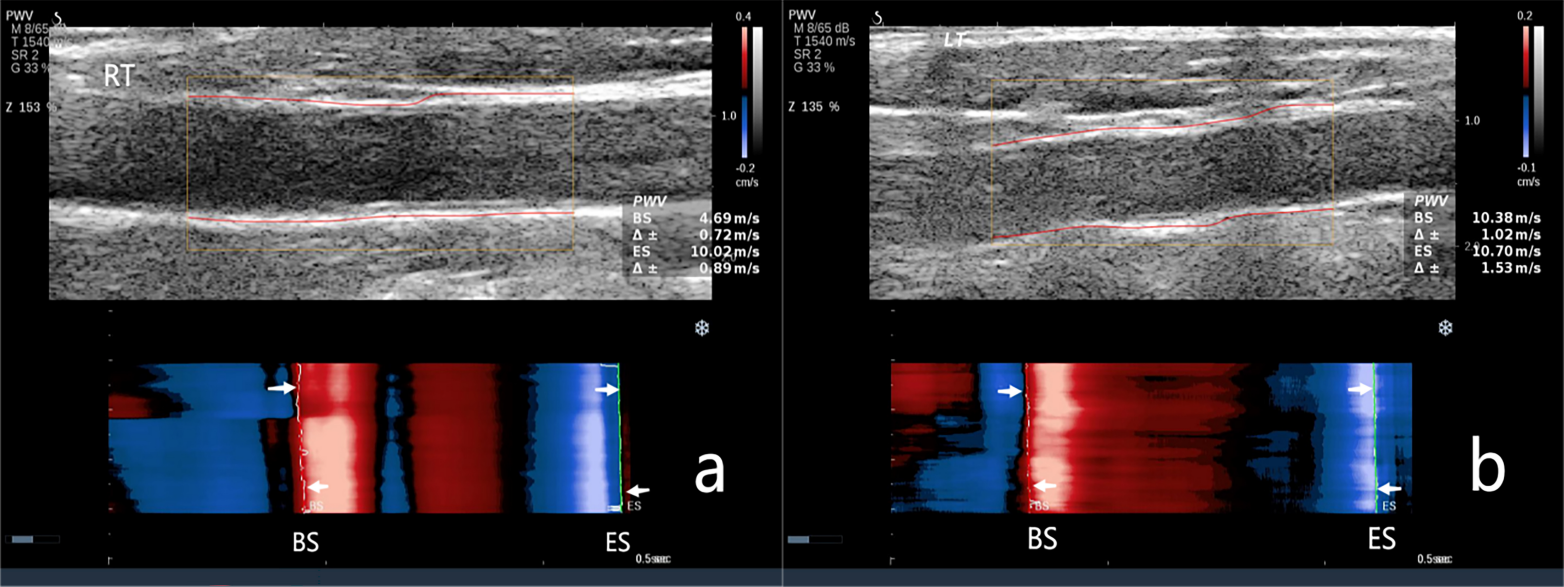
**The carotid artery pulse wave velocity (PWV) in the CAVD group**. (a) PWV measurement of the right carotid artery. PWV-BS, 4.69 m/s; PWV-ES, 10.02 m/s. (b) PWV measurement of the left carotid artery. PWV-BS, 10.38 m/s; PWV-ES, 10.07 m/s. NOTE: PWV-BS corresponds to the slope of the most prominent line in the red band, whereas PWVES is associated with the slope of the most prominent line in the blue band. These features are highlighted by the white arrows in the figure.

The observation group showed higher CIMT and PWV than the normal group (*p* < 0.001) (Fig. 1). Fig. 2 shows the carotid artery PWV for the Non-CAVD group, while Fig. 3 shows the carotid artery PWV for the CAVD group. As the degree of aortic valve calcification increased, the PWV gradually increased; significant differences were observed for the PWV-BS and PWV-ES among all groups (Group One–Three); however, no significant difference was found between Group One and Group Two. Comparatively, significant differences were noted between Group One and Group Two for the PWV-ES and between Group One and Group Three; however, no significant difference was observed between Groups Two and Three. Additionally, there were no significant differences in IMT values across the three groups (Table 2).

**Table 2.** **Comparative analysis of carotid artery IMT, PWV-BS, and PWV-ES among patients with varying degrees of calcification in the CAVD group (n = 105)**.

Group	Case	IMT	PWV-BS	PWV-ES
Group One	48	0.79 ± 0.069	6.81 ± 1.108^a^	9.36 ± 0.769^a^
Group Two	35	0.80 ± 0.068	7.32 ± 0.912^a^	10.93 ± 0.898^b^
Group Three	22	0.79 ± 0.067	8.02 ± 1.024^b^	11.2 ± 0.067^b^
F-value		0.10	10.75	46.72
*p*-value		0.904	<0.001	<0.001

Abbreviations: IMT, intima-media thickness. Different superscript letters (a, b) indicate that the differences between groups are statistically significant (*p* < 005). There was no significant difference among groups with the same letters.

### 3.3 Factors Correlated with the Development of CAVD, PWV-BS, and PWV-ES

Coronary atherosclerotic heart disease and PWV-BS were all significant independent risk factors for CAVD (all *p* < 0.05) (Table 3). The risk factors for PWV-BS included Hypertension, Coronary atherosclerotic heart disease, total cholesterol and homocysteine (*p* < 0.05 for all) (Table 4). The risk factors associated with PWV-ES include Hypertension, Coronary atherosclerotic heart disease, total cholesterol and glycated hemoglobin (*p* < 0.05 for all) (Table 5).

**Table 3.** **Stepwise multivariate binary logistic regression analysis to investigate the factors that influence CAVD (n = 210)**.

	B-value	SE	χ^2^ value	*p*-value	OR value	95% CI
CHD	1.91	0.5	14.64	<0.001	6.74	(2.54–17.92)
PWV-BS	1.20	0.2	34.44	<0.001	3.31	(2.42–4.93)

Abbreviations: CHD, coronary atherosclerotic heart disease; OR, odds ratio.

**Table 4.** **Multivariate linear regression analysis of PWV-BS and clinical indicators (n = 210)**.

	β	SE	t	*p*-value	β (95% CI)
Hypertension	0.90	0.18	5.06	<0.001	(0.55–1.25)
CHD	0.70	0.24	2.89	0.004	(0.22–1.18)
TC	0.17	0.07	2.54	0.012	(0.04–0.30)
HCY	0.03	0.01	2.57	0.011	(0.01–0.05)

**Table 5.** **Multivariate linear regression analysis of PWV-ES and clinical indicators (n = 210)**.

	β	SE	t	*p*-value	(95% CI)
Hypertension	1.01	0.22	4.56	<0.001	(0.57–1.44)
CHD	1.43	0.30	4.77	<0.001	(0.84–2.02)
HbA1c	0.15	0.05	2.89	0.004	(0.05–0.25)
TC	0.25	0.1	2.56	0.011	(0.06–0.44)

## 4. Discussion

CAVD is characterized by progressive fibrous calcification of the aortic valve leaflets, leading to deformity and impaired valve opening and closing. This eventually results in aortic stenosis and/or regurgitation, as well as hemodynamic alterations [14]. In the initial stage, the pathogenesis resembles atherosclerosis, with previous studies demonstrating a correlation between endothelial dysfunction and the development of heart valve sclerosis [15]. An inflammatory response, aberrant calcium and phosphorus metabolism, and oxidative stress are pivotal in valve calcification progression. These mechanisms facilitate calcium deposition within valve tissues and significantly influence arterial elasticity. For example, the inflammatory response exacerbates valve cell damage and fibrosis by activating immune cells and releasing proinflammatory cytokines, including interleukin-6 (IL-6) and tumor necrosis factor-alpha (TNF-α), ultimately contributing to calcium salt accumulation. This response can stimulate the proliferation and migration of vascular smooth muscle cells, further compromising the structural integrity of the arterial wall and reducing its elasticity [16].

In the present study, CAVD was influenced by various factors, including C-peptide, free fatty acids and total cholesterol levels. These factors exhibited a positive correlation with CAVD risk, consistent with the findings of an analysis of factors influencing heart valve calcification by Small AM *et al.* [17]. This current study showed a higher prevalence of hypertension in patients from the observation group compared to those in the Non-CAVD group. Hypertension may contribute to increased pressure on the aortic valve and alterations in shear forces on blood vessels, which may be causally linked. Moreover, direct calcification of the aortic valve can increase the workload on the myocardium. Therefore, prolonged exposure of the aortic valve to elevated pressure can potentially increase calcification.

PWV-BS and PWV-ES were associated with risk factors such as hypertension, coronary atherosclerotic heart disease and total cholesterol (*p* < 0.05 for all). These risk factors indicate that vascular endothelial dysfunction is influenced by diverse pathways, which subsequently influence PWV-BS and PWV-ES. Our research also revealed that lipid metabolism is an independent factor influencing carotid artery elasticity. Abnormal lipid metabolism can lead to endothelial dysfunction, thereby inducing vascular endothelial cell permeability alterations. Consequently, this facilitates the infiltration of low-density lipoprotein particles into the vessel wall, leading to compromised arterial wall elasticity and increased rigidity. The results of the present study concur with previous research findings on atherosclerosis reported by Libby *et al*. [18]. However, in this study the CAVD group exhibited a significantly lower prevalence of Hyperlipidemia compared to the non-CAVD group. This may be attributed to the single-center nature of this study, along with the unique characteristics of the CAVD population and the relatively limited sample size.

In this study, it is significant differences were observed between the observation group and the Non-CAVD group regarding free fatty acids and total cholesterol (*p* < 0.001). Free fatty acids (FFA) is an intermediate product of lipid metabolism. It is well known that serum FFA levels correlate with significant increases in inflammatory responses, oxidative stress, insulin resistance, endothelial dysfunction, and cardiovascular events. This series of mechanisms is closely associated with valve calcification [19] and is a well-established finding reported in numerous studies, thus indicating the potential use of FFA as a clinical predictor for cardiovascular injury.

The carotid artery IMT was within the normal range for all subjects in this study. However, the CAVD group exhibited a significantly higher IMT than the Non-CAVD group (*p* < 0.001). Furthermore, increased valve calcification was associated with significant increases in both the PWV-BS and PWV-ES (*p* < 0.001), indicating subtle alterations in the structural integrity of the carotid artery wall among individuals with CAVD. Despite the increased severity of CAVD, no statistically significant changes were observed in CIMT. While the morphological characteristics of the carotid intima-media remain largely unchanged, significant changes were noted in the elastic properties of the carotid artery. Consequently, it is important to conduct further investigations into the early changes in carotid artery elasticity among patients with CAVD. The standard deviation of the PWV-ES in the left carotid artery was higher than that in the right carotid artery, indicating a more pronounced degree of arterial sclerosis on the left side. Since changes in vascular elasticity manifest at an early stage. The observed disparity may be attributed to differences in the anatomical structure, whereby the left carotid artery originates directly from the aortic arch. In contrast, the right carotid artery arises from the brachiocephalic trunk. Therefore, bilateral carotid artery anatomy differences could result in disparate shear forces exerted on the corresponding walls. The study by Thrysøe *et al*. [20] into carotid plaque distribution among stroke patients revealed a higher prevalence of plaques within the left carotid artery than on the right side, which agrees with the present research findings.

Our study also revealed that PWV-BS is an independent risk factor for CAVD and exhibits a positive correlation. In clinical practice, PWV-BS is regarded as a superior approach for the early diagnosis and quantitative assessment of arterial stiffness. This aligns with the findings by Zhu *et al*. [21] on carotid stiffness and atherosclerotic risk.

In summary, for individuals in high-risk groups, the regular assessment of carotid PWV can serve as a vital preventive strategy. This diagnostic tool enables healthcare professionals to evaluate an individual’s cardiovascular status more precisely and promptly identify potential risk factors. Conditions such as hypertension, diabetes, and hypercholesterolemia are significant contributors to cardiovascular disease, and alterations in PWV often serve as early indicators of these conditions. Consequently, routine monitoring of carotid PWV facilitates early risk detection and familial predisposition and provides a scientific foundation for subsequent therapeutic interventions. Meanwhile, proactive management and mitigation of recognized risk factors are equally important in addition to regular screening. This includes maintaining a balanced diet, engaging in consistent physical activity, abstaining from smoking, moderating alcohol consumption, and adhering to prescribed medication regimens. A holistic approach to health management can markedly reduce the likelihood of cardiovascular incidents, enhancing overall quality of life.

In summary, adopting a proactive stance toward cardiovascular health issues is an important way to safeguard high-risk individuals. Finally, this study is not without limitations. Indeed, this study was conducted at a single center, and given the unique characteristics of the CAVD population and the limited sample size, some selection bias is inevitable. Therefore, future studies should be multi-center and aim to expand the sample size to overcome this limitation.

## 4. Conclusions

The UFPWV technique can detect early changes in arterial stiffness in patients with CAVD, thereby highlighting the initial development of arteriosclerosis. Based on these identified risk factors, future risk stratification may be performed for carotid artery stiffness patients with CAVD. This study has significant clinical implications for the early prevention, diagnosis, and treatment of arterial stiffness in patients with CAVD. Furthermore, the results significantly impact early diagnosis, control of calcification progression, and mitigation of vascular damage caused by arteriosclerosis to improve prognoses and slow the progression of cardiovascular disease.

## Availability of Data and Materials

Due to privacy considerations, the data for this study are not publicly available. However, the corresponding author can provide the data upon reasonable request.
